# L-Tryptophan Aqueous Systems at Low Concentrations: Interconnection between Self-Organization, Fluorescent and Physicochemical Properties, and Action on Hydrobionts

**DOI:** 10.3390/nano12111792

**Published:** 2022-05-24

**Authors:** Irina S. Ryzhkina, Lyaisan I. Murtazina, Larisa A. Kostina, Diana A. Sharapova, Irina S. Dokuchaeva, Svetlana Yu. Sergeeva, Kristina A. Meleshenko, Andrew M. Petrov

**Affiliations:** 1 Arbuzov Institute of Organic and Physical Chemistry, FRC Kazan Scientific Center, Russian Academy of Sciences, 8 Arbuzov Str., 420088 Kazan, Russia; limurt@yandex.ru (L.I.M.); lar.kostina2116@yandex.ru (L.A.K.); diana777_94@mail.ru (D.A.S.); 183561@mail.ru (I.S.D.); sergeevas@iopc.ru (S.Y.S.); kmeleshenko@inbox.ru (K.A.M.); 2 Institute for Problems of Ecology and Mineral Wealth Use of Tatarstan Academy of Sciences, 28 Daurskaya Str., 420087 Kazan, Russia; zpam2@yandex.ru

**Keywords:** aqueous dispersed systems, *L*-Tryptophan, low concentration, aquatic ecosystem protection, hydrobionts, harmful action

## Abstract

As shown by fluorescence monitoring of dissolved organic matter, amino acid *L*-Trp can be present in natural water. The consequences of the presence of *L*-Trp at low concentrations in surface water systems are not yet established for hydrobionts. Studying the physicochemical patterns, as well as their relationships to the bioeffects of *L*-Trp solutions in the low concentration range, can provide new and important information regarding the unknown effects of *L*-Trp. The self-organization, physicochemical properties, fluorescence, UV absorption, and action of *L*-Trp solutions on *Paramecium caudatum* infusoria, *Chlorella vulgaris* algae were studied in the calculated concentrations range of 1 × 10^−20^–1 × 10^−2^ mol/L. The relationship between these phenomena was established using the certified procedures for monitoring the toxicity of natural water and wastewater. It was shown for the first time that aqueous solutions of *L*-Trp are dispersed systems in which the dispersed phase (nanoassociates) undergoes a rearrangement with dilution, accompanied by coherent changes in the nanoassociates’ parameters and the properties of systems. The non-monotonic concentration dependence of fluorescence intensity (λ_ex_ at 225 nm, λ_em_ at 340 nm) is in good agreement with the data on the nanoassociates’ parameters, as well as with both the physicochemical properties of the systems and their bioassay results.

## 1. Introduction

**Scheme 1:**
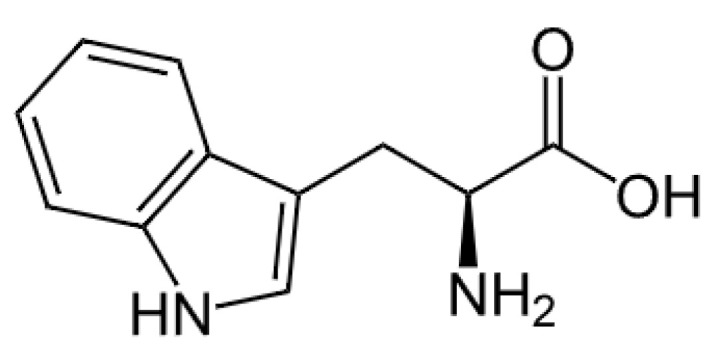
*L*-Trp.

*L*-tryptophan (*L*-Trp) is a proteinogenic-essential heterocyclic aromatic α-amino acid (Scheme 1) that constitutes the proteins of all known animal organisms and plant bodies [1]. One of the most interesting spectral properties of *L*-Trp is that it has pronounced fluorescence, which is mostly explained by a π-system of the indole fragment of the *L*-Trp molecule [2,3].

Since tryptophan residues contribute the most to protein fluorescence and are highly sensitive to a microenvironment, tryptophan fluorescence serves as a basic tool to assess the structural state of biomolecules in solutions and biological fluids [2,3,4,5]. In addition, tryptophan fluorescence is used in surface water quality control [6,7]. As shown by fluorescence monitoring of dissolved organic matter (DOM), *L*-Trp is often present in surface water systems, either as a part of protein-like fractions, or in a free state, or both.

Proteinogenic amino acids, including *L*-Trp, are known to be involved in the regulation of intercellular interactions in organisms of different complexity levels [8,9,10,11]. In this case, the activity of amino acids and their metabolites is usually observed at low concentrations, comparable with nanomolar concentrations of regulatory peptides [12,13]. It was shown that *L*-Trp and its metabolites, *L*-kynurenine, serotonin, and indole-3-acetic acid (heteroauxin), have a regulatory effect in the range of calculated concentrations of 10^−7^–10^−19^ mol/L [9,14,15].

Despite the practical significance and opportunities opened by the application of amino acids in nanoconcentrations, their use in such doses is constrained by insufficient knowledge about the specifics of the physicochemistry of highly diluted systems and their role in the occurrence of the observed bioeffects, as is the case for many other biologically active compounds (BACs) [16,17]. Concentration dependencies of the drug solutions’ bioeffects in the low calculated* concentrations range usually have a complex form and are characterized by a number of features, such as non-monotonic and multidirectional action (i.e., the change in the bioeffect sign), the presence of a “silent zone” where the biosystem is virtually insensitive to the drug effects, etc. [16,17]. (* For the reasoning behind the usage of the “calculated concentration” term please refer to the Sections “2. Materials and Methods” and “2.2. Experimental Design”).

A convenient method for assessing BACs’ bioeffects in the range of low concentrations is to study the effects of their solutions on the growth and development of hydrobionts [18,19,20,21]. Ecotoxicological tests are currently becoming more and more in demand as they are closely related to the pressing issue of aquatic ecosystem protection [22,23,24,25,26,27].

Previously published data suggest that the factors that trigger changes in the ecological state of water bodies, disturbances in food chain links, and degradation of biocoenosis are directly related to the widespread use of medicinal, veterinary, and agricultural chemicals that are present in water in low environmentally significant concentrations within the 10^6^ ng/L–10^−2^ ng/L range [18,20,21,22,24,25,26]. Some pesticides, pharmaceuticals, and biogenic substances were found to cause damage to hydrobionts at similar and much lower calculated concentrations (1 × 10^−14^–1 × 10^−18^ mol/L) [20,21,24]. The consequences of the *L*-Trp presence in natural water at low concentrations are not yet established for aquatic organisms and humans. Studying the physicochemical and spectral patterns of diluted *L*-Trp solutions, as well as their relation to the solutions’ bioeffects, can provide conceptually new and important information regarding the unknown effects of diluted aqueous solutions and help to develop the natural water diagnostic methods, while improving the further understanding of the origin and composition of DOM [6,7,28].

A physicochemical explanation of the bioeffects of highly diluted BACs’ solutions has appeared relatively recently. It has been shown that highly diluted aqueous solutions of many BACs are open self-organized disperse systems formed with the involvement of water structures, capable of the disperse phase rearrangement (domain-nanoassociate, nanoassociate-nanoassociate types). The parameters of the disperse phase change non-monotonically with dilution, which is reflected in a coherent change in the physicochemical and biological properties of the systems [20,29]. One of the main differences between domains and nanoassociates is that domains are usually formed at sufficiently high concentrations of the substance (1–10^−5^ mol/L) in both the presence and absence of background low-frequency electromagnetic fields (EMF). On the other hand, nanoassociates only form in the presence of EMF as the solution is diluted further. They are characterized by a size (d) of hundreds of nm and a negatively charged interface [29]. It was found that the formation and rearrangement of domains and nanoassociates are also accompanied by the appearance and non-monotonic change in UV absorption in the region of 200–300 nm [20,21].

Works [28,30,31,32,33,34,35] also show the presence of similar absorption bands in the UV spectra of natural water, diluted aqueous solutions of some BACs, and systems containing structured water (EZ-water) in contact with a hydrophilic surface. Excitation in this UV region causes fluorescence in two spectral ranges of 300–350 and 400–450 nm. The obtained results allowed the authors [30,31,32,33,34,35] to assume that the observed spectral properties are a characteristic of the ordered aqueous structures formed in nanoheterogeneous aqueous systems. To support this assumption, similar fluorescence spectra of highly diluted dispersed systems based on some BACs that do not have a pronounced emission capability were obtained [36,37,38,39].

To the best of our knowledge, despite the available experimental material concerning the bioeffects of diluted solutions of *L*-Trp and its metabolites, no attempt has been made so far to relate these bioeffects to the self-organization, physicochemical, and spectral properties of *L*-Trp systems at low concentrations, nor to investigate the possible affiliation of *L*-Trp and BACs-diluted aqueous systems with the presence of a tryptophan-like band in natural water’s fluorescence spectra.

Thus, the aim of this work is to study the self-organization, physicochemical properties, UV absorption, and fluorescence properties of *L*-Trp systems in the range of calculated concentrations 1 × 10^−20^–1 × 10^−2^ mol/L along with their impact on the growth and development of aquatic biota—*Chlorella vulgaris* single-celled green algae, *Paramecium caudatum* infusoria—as well as establishing the relationship between these phenomena using methods of dynamic and electrophoretic light scattering, conductometry, pH-metry, tensiometry, and UV and fluorescence spectroscopy, together with certified techniques for monitoring the toxicity of natural and waste waters.

## 2. Materials and Methods

### 2.1. Chemicals

*L*-Trp (purity > 99.5%) was purchased from Sigma-Aldrich, China. Solutions were prepared in 15 mL Wiegand (120011543) vials using only freshly prepared double-distilled water with specific conductivity of no more than 1.5 μS/cm and free of any particles, as checked by a Malvern Instruments Zetasizer Nano ZSP analyzer.

### 2.2. Experimental Design

The initial substrate’s solution of 1 × 10^−2^ mol/L concentration was prepared from *L*-Trp diluted with de-ionized water. Sample solutions (10 mL) were prepared via sequential decimal dilution, stirred using a minishaker (Shaker lab dancer, IKA, Staufen, Germany) for 10 s after dilution and kept for 20 h on the laboratory bench. In the case of high dilutions (less than 1 × 10^−12^ mol/L), the actual concentration of the substance is rather difficult to confirm by experimental methods. Therefore, in our previous papers [29,36,37,38,39], as well as in the present work, when discussing high dilutions corresponding to calculated concentrations from 1 × 10^−13^ mol/L to 1 × 10^−20^ mol/L, we use this very term implying the theoretically possible concentration of the substance at a corresponding dilution step.

The method of analysis of highly diluted solutions involves the study of self-organization and properties of such solutions in two parallel series [29]. The difference between the first and the second series is that in the first one the sample solutions are kept on the laboratory bench under ambient conditions before being studied by the physicochemical methods, and in the second they are kept in a cylindrical three-layer permalloy container that protects the contents from external electromagnetic fields (EMFs) with screening coefficients of ~1000 (hypomagnetic conditions). Using this method, it is possible to establish a threshold concentration (c_thr_), below which structures formed in solutions are called nanoassociates, and above which are called molecular-water domains [29]. Prior to the measurements, the solutions were kept at a constant temperature of 25 ± 0.1 °C for 1 h [29].

### 2.3. Physicochemical Methods

#### 2.3.1. Conductometry

Changes in the electrical conductivity (χ) of the solutions at 25 ± 0.1 °C were determined using a conductometer (inoLab Cond Level 1, WTW, Weilheim, Germany).

#### 2.3.2. pH

pH was measured by a pH-meter (inoLab pH 720, WTW, Weilheim, Germany) at 25 ± 0.1 °C.

#### 2.3.3. Tensiometry

Surface tension (σ) of the solutions at 25 ± 0.1 °C was determined using a highly sensitive tensiometer (Sigma 720 ET, KSV Instruments, Helsinki, Finland).

#### 2.3.4. Dynamic Light Scattering (DLS)

The particle size (the effective hydrodynamic diameter (d) of kinetically labile particles at the maximum of the distribution curve) was determined on a Zetasizer Nano ZSP analyzer (Malvern Instruments, Malvern, Worcestershire, UK) equipped with a 633 nm He–Ne laser and operating at an angle of 173°. The optical configuration using a 173° detection angle makes it possible to measure samples with low concentrations and particle sizes in the range from 0.6 nm to 6000 nm. The data were collected and analyzed using the Dispersion Technology Software version 7.10 (Malvern). The main advantages of this software are listed at [40]. The combination of these factors results in the exceptional sensitivity of the Zetasizer Nano ZSP analyzer, which is essential for the measurement of small nanoparticle sizes at low concentrations. In the case of diluted open self-organized systems, such as the studied *L*-Trp systems, the practical detection limit of the Zetasizer Nano ZSP analyzer is determined by the type of correlation function and polydispersity index automatically provided by the analyzer to the user. Each sample was measured in single-use polystyrene cuvettes (Sarstedt, Germany) with a pathlength of 10 mm. The measurements were made at a position of 4.65 mm distance from the cuvette wall with an automatic attenuator and at a controlled temperature of 25 ± 0.1 °C.

#### 2.3.5. Electrophoretic Light Scattering (ELS)

The ζ-potential of the *L*-Trp systems was determined on a Zetasizer Nano ZSP analyzer (Malvern Instruments Ltd., Malvern, Worcestershire, UK). Each sample was measured in a single-use U-shaped capillary cell (DTS 1071, Malvern Instruments Ltd., Malvern, Worcestershire, UK) at a controlled temperature of 25 ± 0.1 °C.

During the DLS and ELS methods investigations, the solutions were freed of dust by filtering through Iso-Disc N-25-4 Nylon (Supelco, Bellefonte, PA, USA) filters. Each point shown in concentration dependencies of size and ζ-potential is a result of statistical processing of data obtained by measuring the size or ζ-potential of three parallel samples of the same concentration. Each sample was measured six times. Then we used Microsoft Excel to find the arithmetic mean of the size or ζ-potential measurement and the standard deviation of the measurement. Concentration dependences show the arithmetic mean with the standard deviation for each measurement.

Statistical processing of the results was carried out by parametric statistics using Microsoft Excel with a statistical reliability of 95%. The measurement errors of nanoassociate parameters and physicochemical properties of solutions (specific electrical conductivity, pH, surface tension) were in the range of 2–20%.

#### 2.3.6. UV–Vis Spectroscopy

The UV absorption spectra for the *L*-Trp systems in the range of calculated concentrations from 1 × 10^−20^ mol/L to 1 × 10^−4^ mol/L were obtained using a UV/Vis Cary 100 spectrophotometer (Agilent Technologies, Santa Clara, CA, USA). This is a double beam spectrophotometer with Czerny–Turner monochromator, a wavelength range of 190–900 nm and a resolution of 0.1 nm. The accuracy and reproducibility of wavelengths were <±0.02 and <0.008 nm, respectively; the scanning rate was equal to 600 nm/min with an interval of 1 nm. We used QS-SUPRASIL quartz cells 10 mm in length. The UV absorption spectra of the *L*-Trp systems, obtained by excluding the baseline of the water used to prepare the solutions, were reproduced many times; the differences in the spectra of parallel-prepared samples were insignificant. The obtained data were analyzed using Microsoft Excel and Origin Pro 2015 software.

#### 2.3.7. Fluorescence Spectroscopy

Fluorescence spectra were recorded using a Cary Eclipse Fluorescence Spectrophotometer (Agilent Technologies, Santa Clara, CA, USA). It is equipped with a xenon lamp and two Czerny–Turner monochromators. The slits on the excitation and emission monochromators had widths of 5 nm. The detector voltage was high. Standard (10 mm) quartz fluorescence cells (Part No. 6610000900, Agilent Technologies, Waldbronn, Germany) were used. After their loading, we enabled a 10 min thermal equilibration. The cells were thermostated using a Peltier element. The emission and excitation spectra of the *L*-Trp systems in the concentration range from 1 × 10^−4^ mol/L to 1 × 10^−6^ mol/L were obtained with the “medium voltage” detector setting, and for the range of 1 × 10^−7^ mol/L and below with the “high voltage” detector setting.

We studied fluorescence spectra at excitation wavelengths (λ_ex_) of 225 nm, as well as excitation spectra at an emission wavelength (λ_em_) of 340 nm. The concentration dependencies of fluorescence intensity show the standard deviation obtained by processing the intensity (λ_em_ 340 nm) from three measurements of the same sample. The discrepancy between the results of the three parallel experiments did not exceed 20%. In cases where the shape of the fluorescence band changed, the given values represent an estimate. The obtained data were analyzed using Microsoft Excel and Origin Pro 2015 software (Northampton, MA, USA).

### 2.4. Toxicological Methods

The toxic action of the *L*-Trp systems on algae (*Chlorella vulgaris*) and infusoria (*Paramecium caudatum*) was tested using the certified procedures for the monitoring of toxicity of natural waters and wastewater, which are detailed in [21].

#### 2.4.1. Biotesting on Infusoria *Paramecium caudatum*

The method was based on the determination of the number of infusoria (*P. caudatum*) when exposed to toxic substances present in the water sample under study, compared to the control. The determination of the toxicity of each sample was carried out in five parallel series. Five parallel batches with cultivation water (dechlorinated water, pH = 7.0–8.0, t = (20 ± 2) °C) were used as a control. For biotesting, a micro-aquarium with wells was used, which was placed on the stage of a stereomicroscope. Ten to twelve individuals were placed in each well; five wells were used for control and five wells for a solution of a certain concentration, the toxicity of which was being determined. Then, 0.6 mL of cultivation water was poured into the control wells, and 0.6 mL of the test solution was added to the experimental wells. The micro-aquarium was placed in a thermostat and kept for 24 h at a temperature of 22–24 °C. Next, the numbers of individuals were counted under the microscope.

To assess the acute toxicity of a sample, the percentage change in the number of paramecium (A, %) was calculated by Equation (1):

(1) $$A=\frac{X_{d}}{X_{i}}\times 100\%$$

where X_i_ and X_d_ are the number of individuals initially and after 24 h, respectively.

The acute toxicity of *L*-trp solutions on infusoria (*P. caudatum*) was defined as a change of at least 50% in the number of infusoria during 24 h. The concentration of *L*-Trp was considered harmless (not leading to an acute toxic effect) if the change in number of infusoria did not exceed 10% during 24 h.

#### 2.4.2. Biotesting on *Chlorella vulgaris* Green Algae

The method was based on registering differences in the optical density of the test culture of the *C. vulgaris* green algae grown on a medium that did not contain toxic substances (control) and in the studied solutions.

In total, 2 mL of the test culture in 50% Tamiya medium containing 12.5 × 10^6^ cells/mL was introduced into 48 mL glass vessels containing control and test solutions. The contents of each glass were mixed and 6 mL of the mixture was then poured in four reactors, which were then placed in a multi-cell cultivator KVM-05 (T = (36 ± 0.5) °C, light intensity 60 W/m^2^, hold-up time 22 h). After 22 h, the optical density (λ = 560 nm) was measured in each reactor.

The relative difference in the average optical density (l, %) for each dilution compared with the control was calculated by Equation (2):

(2) $$l=\frac{D_{c}-D_{t}}{D_{c}}\times 100\%$$

where D_c_ and D_t_ are average values of optical density in the control and test solution, respectively.

The toxicity criterion on algae tests was at least 20% change during 22 h in the optical density level because of inhibition or stimulation of the algae sample with respect to the reference.

## 3. Results and Discussion

**Figure 1:**
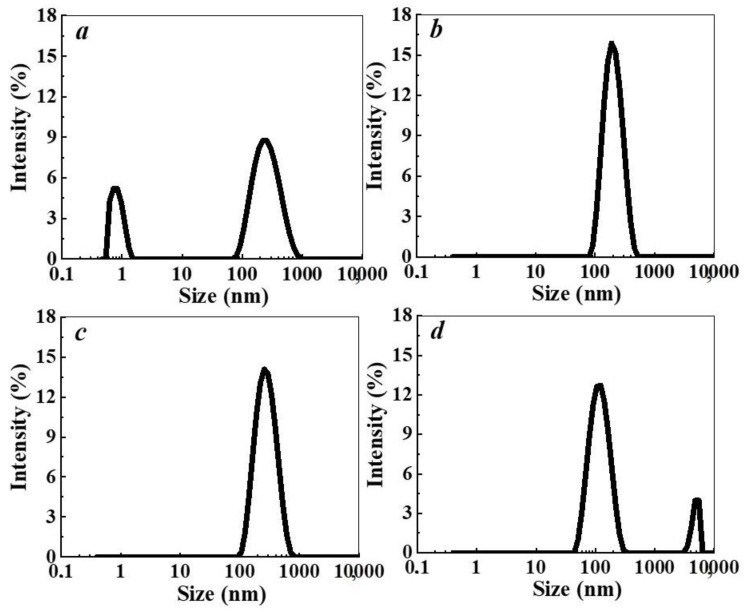
Particle size distribution based on light scattering intensity in the *L*-Trp systems. Concentration (c, mol/L) of *L*-Trp as follows: (**a**) 1 × 10^−2^, (**b**) 1 × 10^−4^, (**c**) 1 × 10^−11^, (**d**) 1 × 10^−16^ mol/L, 25 °C.

The dynamic light-scattering (DLS) study of the *L*-Trp aqueous solutions in a wide range of calculated concentrations, 1 × 10^−20^–1 × 10^−2^ mol/L, showed that they are self-organized disperse systems, in which the disperse phase of a different nature is formed with dilution (Figure 1a–d). Such behavior is characteristic for aqueous solutions of many BACs [29].

At the concentration of 1 × 10^−2^ mol/L, bimodal particle-size distribution of the light-scattering intensity indicates the presence of “micelle-like” particles [41] and domains [41,42,43] being about 1 nm and hundreds of nanometers in size, respectively (Figure 1a). In contrast to the particle-size distribution of the light-scattering intensity, the volume particle-size distribution indicates that only particles of about 1 nm in size form in the system at 1 × 10^−2^ mol/L. The correlation functions and volume particle-size distributions are given in Supplementary Materials Figure S1. According to [44], the obtained result suggests that in this case the number of domains is small compared to the micelle-like particles. Upon dilution up to 1 × 10^−3^ mol/L, the content of micelle-like particles decreases, while that of the domains increases, which is typical for most BACs solutions [29].

Starting from 1 × 10^−4^ mol/L and below (Figure 1b–d), there is a predominant formation of a dispersed phase with a size of hundreds of nanometers consisting of the substance molecules and ordered water structures [41,42,43,45], which undergoes domain-nanoassociate, nanoassociate-nanoassociate rearrangement as dilution proceeds. An example of the correlation functions and volume particle size distributions in this concentration range are given in Supplementary Materials Figure S2.

Keeping solutions in a permalloy container that shields low-frequency EMFs, similarly to [29], shows that for *L*-Trp systems the threshold concentration (c_th_) is 1 × 10^−7^ mol/L. Thus, for the *L*-Trp systems, we can arbitrarily divide the low concentration range into two intervals, characterized by the formation of domains (1 × 10^−7^ to 1 × 10^−4^ mol/L) and nanoassociates (1 × 10^−2^ to 1 × 10^−8^ mol/L). Significant changes in the properties of self-organized systems are often observed in the vicinity of c_th_ [29,36,37,38,39].

**Figure 2:**
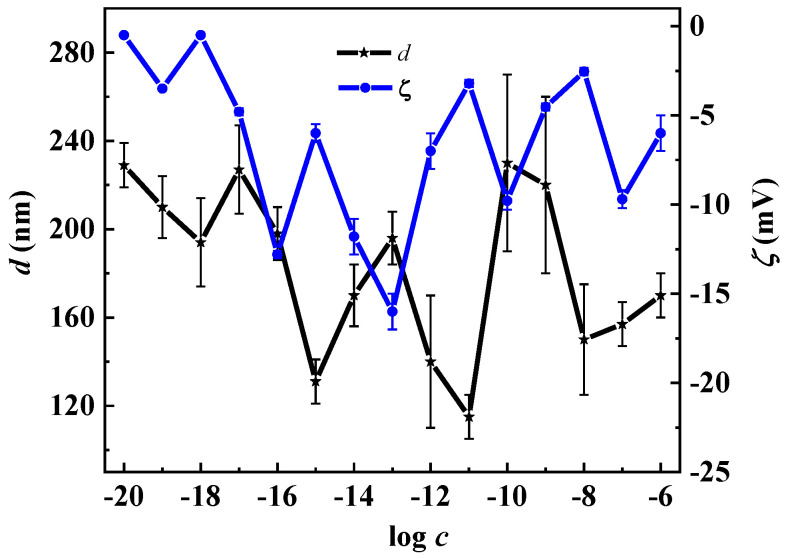
Dependence of the size of particles (d) and ζ-potential (ζ) on the concentration (c, mol/L) of the *L*-Trp systems, 25 °C.

Studying the tryptophan solutions by the electrophoretic light-scattering (ELS) method, we found that the average ζ-potential of micelle-like particles is −13.0 ± −2.0 mV, domains −8.0 ± −1.5 mV, and for nanoassociates these values change non-monotonically from −1.0 mV to −16.0 mV (Figure 2). The negative ζ-potential of micelle-like particles and domains is most likely due to the fact that they are formed mainly by *L*-Trp zwitterions. Nanoassociates’ common characteristic feature is a negative sign of their ζ-potential, which is independent of the molecular structure or the charge of the BACs ions. The mechanism underlying this phenomenon has not yet been discovered, but according to the works [45,46], it may be related to the properties of the ordered water structures that form a nanoassociate. Figure 2 shows the non-monotonic-correlated concentration dependencies of the nanoassociates’ size (d) and ζ-potential. A consistent increase of both parameters is observed at concentrations of 1 × 10^−17^, 1 × 10^−13^ and 1 × 10^−10^ mol/L, while a correlated decrease is seen at concentrations of 1 × 10^−18^, 1 × 10^−15^, and 1 × 10^−11^ mol/L.

**Figure 3:**
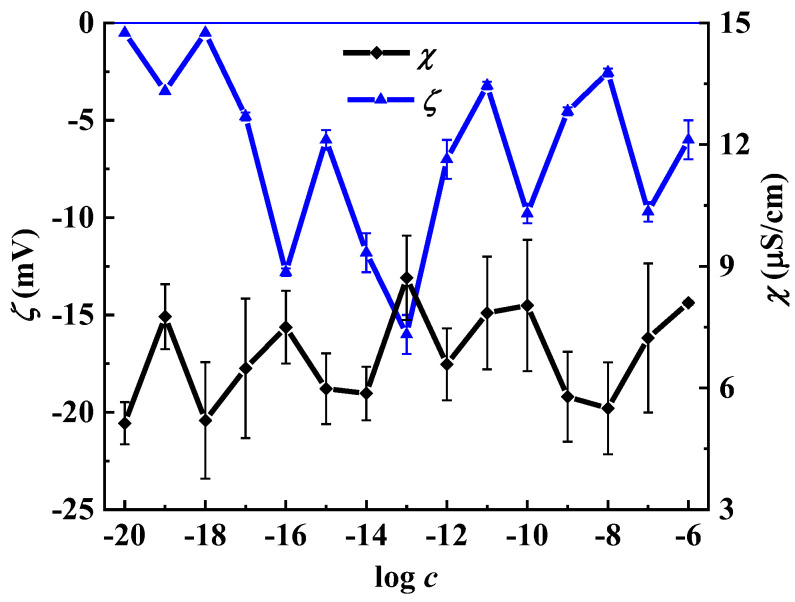
Dependence of the specific conductivity (χ) and ζ-potential (ζ) on the concentration (c, mol/L) of the *L*-Trp systems, 25 °C.

It is known that the formation and rearrangement of the dispersed phase initiates the emergence of non-monotonic concentration dependencies of systems’ physicochemical properties [29]. Figure 3 and Figure S3 (Supplementary Materials) show non-monotonic concentration dependencies of the ζ-potential (Figure 3) and d (Figure S3) of nanoassociates and specific conductivity (χ) values of the *L*-Trp systems. It is clearly seen that not only the nanoassociates’ parameters but also the systems’ χ reach their extreme values at the abovementioned concentrations or in their vicinity.

**Figure 4:**
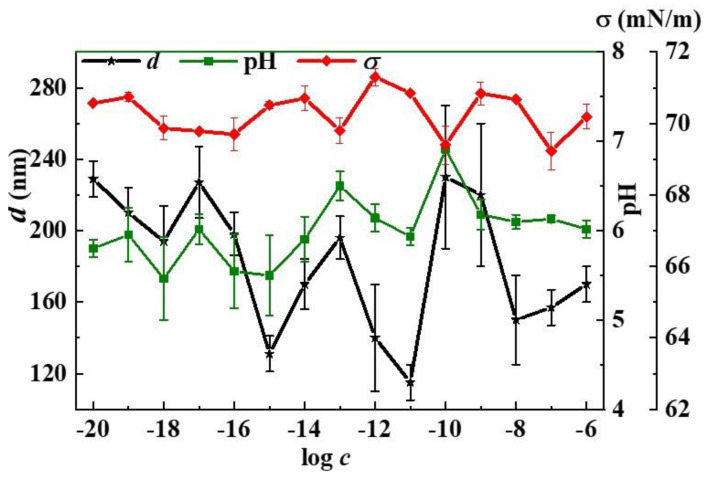
Dependence of the size of particles (d), pH, and surface tension (σ) on the concentration (c, mol/L) of the *L*-Trp systems, 25 °C.

Figure 4 shows non-monotonic concentration dependencies of nanoassociates’ d and the systems’ pH and surface tension (σ) values. Both this figure and Figure 3 clearly demonstrate the relationship of the dependencies of nanoassociates’ parameters and systems’ properties, which change in phase (d and pH) or in antiphase (d and σ, ζ-potential and χ) with extremes in the vicinity of critical concentrations 1 × 10^−17^, 1 × 10^−13^, and 1 × 10^−10^ mol/L.

The surface tension isotherm of the *L*-Trp systems in a wider concentration range 1 × 10^−20^–1 × 10^−2^ mol/L (Supplementary Materials, Figure S4) presents a non-monotonic decrease in σ not only in the range of nanoassociates formation (1 × 10^−20^ to 1 × 10^−8^ mol/L), but that of domains as well (1 × 10^−7^–1 × 10^−4^ mol/L), with minimum values at the threshold concentration of 1 × 10^−7^ mol/L and at 1 × 10^−5^ mol/L. Works [29,38,39] relate a local decrease of surface tension at certain low concentrations of BACs to the possibility of more pronounced bioeffects at these concentrations, as surface tension is responsible for membranotropic properties of disperse systems, what will be discussed in more detail below.

Thus, the rearrangement of domains and nanoassociates in self-organized *L*-Trp systems is accompanied by a consistent non-monotonic change of the disperse phase parameters and of the physicochemical properties—specific conductivity, pH, and surface tension—reaching extreme values at the same critical concentrations.

**Figure 5:**
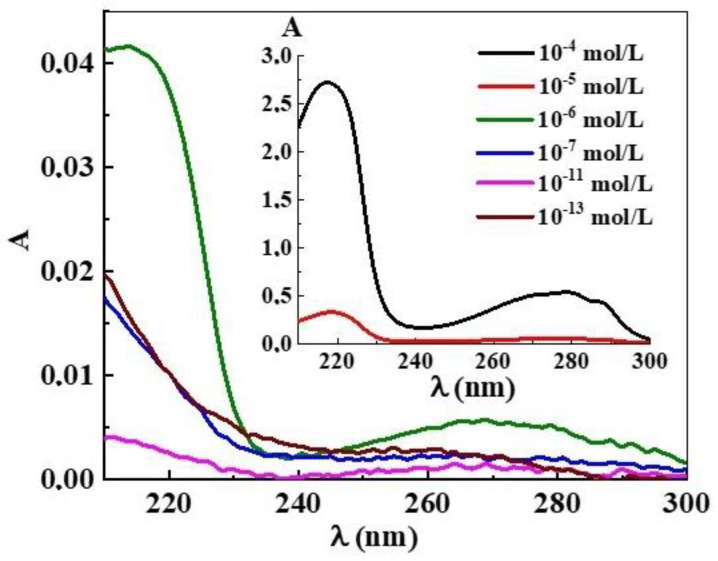
Absorption spectra of the *L*-Trp systems, 25 °C. Insert: Absorption spectra of the *L*-Trp systems with concentrations of 1 × 10^−4^ and 1 × 10^−5^ mol/L.

UV absorption (A) spectra of *L*-Trp systems in the range from 1 × 10^−20^ mol/L to 1 × 10–4 mol/L were obtained after deducting the baseline of the water used to prepare the solutions (Figure 5). In the vicinity of the threshold concentration of 1 × 10^−6^ mol/L, the spectrum shape of the system is close to that of the *L*-Trp systems at a higher concentration range of 1 × 10^−5^–1 × 10^−4^ mol/L [2,3], with maxima at 218 nm and 270–280 nm (Figure 5, inset). Starting from the threshold concentration of 1 × 10^−7^ mol/L and lower, the spectra have a different appearance characteristic of the previously described BACs systems in similar low concentration ranges where nanoassociates are formed, with a weakly pronounced shoulder at 225 nm and a band in the 250–270 nm interval [20,36,37,38,39].

Careful measurements of the UV spectra of progressively diluted *L*-Trp systems (Figure 5) show the presence of small but clear differences of absorption curves in the region of the A_225_ shoulder. The changes of A_225_ are non-monotonic in nature. The obtained data are consistent with the results published in [20,36,37,38,39], where it was found that the non-monotonic change in A_225_ upon the systems’ dilution is associated with changes in the nanoassociate parameters.

**Figure 6:**
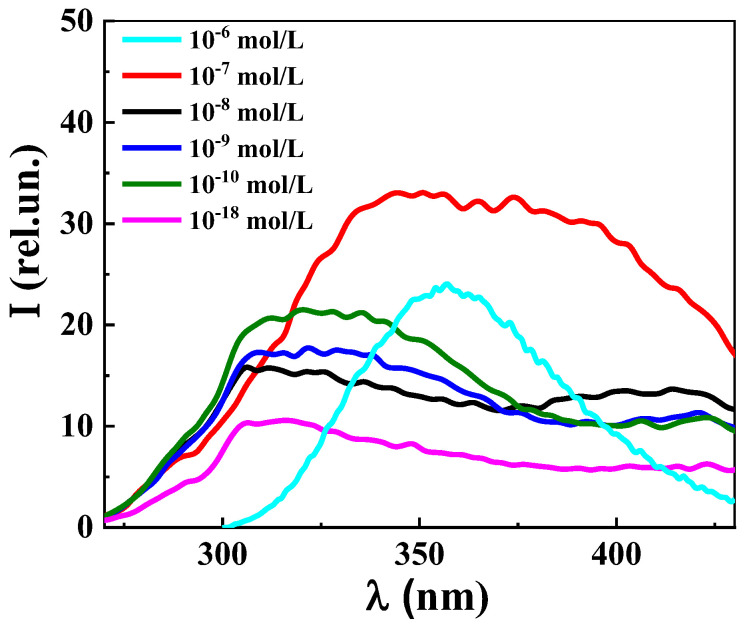
Fluorescence spectra (λ_ex_ 225 nm) of *L*-Trp systems, 25 °C. At 1 × 10^−6^ mol/L, medium voltage was used; at 1 × 10^−7^ mol/L and below, high voltage.

Figure 6 shows the fluorescence spectra (λ_ex_ 225 nm) of *L*-Trp systems with concentrations of 1 × 10^−6^, 1 × 10^−7^, 1 × 10^−8^, 1 × 10^−9^, 1 × 10^−10^, and 1 × 10^−18^ mol/L obtained in the “medium voltage” mode (1 × 10^−6^ mol/L) and “high voltage” mode (1 × 10^−7^ mol/L and below). Starting from the threshold concentration of 1 × 10^−7^ mol/L, the spectrum shape becomes different from the spectra of *L*-Trp systems at concentrations of 1 × 10^−6^–1 × 10^−4^ mol/L (Figure S5 and Figure 6), the latter being characterized by an almost symmetrical band in the 300–400 nm range with a maximum at 350–360 nm analogous to [2,3]. The system’s spectrum at 1 × 10^−7^ mol/L is a wider band at a 290–440 nm range, which has a plateau with a maximum at 340–400 nm. Apparently, this is a complex band made up from an *L*-Trp band and two bands characteristic of the more diluted BACs systems in which nanoassociates are formed [36,37,38,39].

In the concentration range 1 × 10^−20^ to 1 × 10^−8^ mol/L of the *L*-Trp systems, the fluorescence spectra (λ_ex_ 225 nm) show two broad overlapping bands in the two spectral ranges of 290–375 and 375–440 nm. The emission spectrum of the *L*-Trp system at a concentration of 1 × 10^−8^ mol/L, located at the boundary of domains-nanoassociates restructuring, has a short-wave band with a maximum of 310–340 (band 310–340) and a long-wave band with a maximum at 420 (band 420), which has a slightly less intensity than the 310–340 nm band, similarly to [36,37,38,39]. The maximum of the 420 band practically disappears upon dilution in the interval 1 × 10^−12^–1 × 10^−18^ mol/L, its intensity becoming approximately two times lower than that of the 310–340 band, which is also characteristic of the emission spectra of diluted systems of BACs [36,37,38,39].

The obtained data indicate that as the concentration decreases in the range 1 × 10^−20^–1 × 10^−8^ mol/L, the 310–340 band (λ_ex_ 225 nm) shows a non-monotonic change in its intensity, while maintaining its overall shape, which according to [36,37,38,39] suggests that the 310–340 band in *L*-Trp systems, similarly to other highly diluted BACs solutions, is associated with the formation and rearrangement of nanoassociates.

This conclusion is consistent with the results reported in the work [47], which describes the discovery of a short-wave shoulder with a maximum around 300 nm on the fluorescence spectrum of an ethanol-water solution of *L*-Trp. According to the authors, this band is related to the structure-formation processes taking place in ethanol–water solutions of *L*-Trp, making it possible to use fluorescence as a marker when studying the dynamics of supramolecular ensembles’ emergence and destruction.

**Figure 7:**
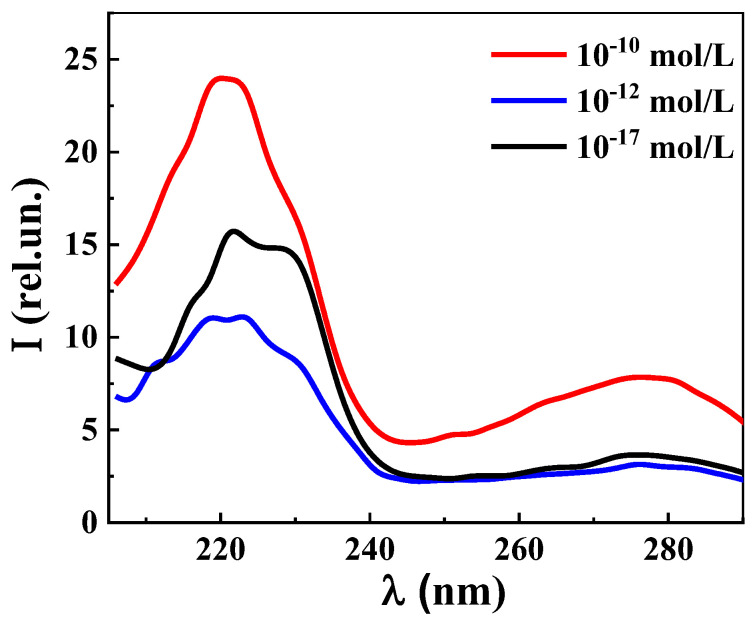
Excitation spectra (λ_em_ 340 nm) of *L*-Trp systems, 25 °C.

Figure 7 shows the excitation spectra (λ_em_ 340 nm) of the *L*-Trp systems at concentrations of 1 × 10^−10^, 1 × 10^−12^, and 1 × 10^−17^ mol/L. The spectra have the form of two weakly overlapping broad bands in the short-wave (215–240 nm) and long-wave regions (260–290 nm). It is apparent that the short-wave band with a maximum at 220 nm is approximately three times more intense than the long-wave band with a maximum at 275 nm. Excitation and absorption spectra (Figure 5) are close, which is typical for fluorescent systems [2,3]. The excitation spectra of the *L*-Trp systems are similar to those of the previously studied diluted BACs systems [36,37,38,39].

The results stated above allow us to conclude that in the concentration range below 1 × 10^−7^ mol/L, the observed fluorescence of *L*-Trp systems, as is the case for other diluted BACs systems [36,37,38,39], is related to the formation and rearrangement of nanoassociates consisting mainly of ordered water structures [29,45,46].

**Figure 8:**
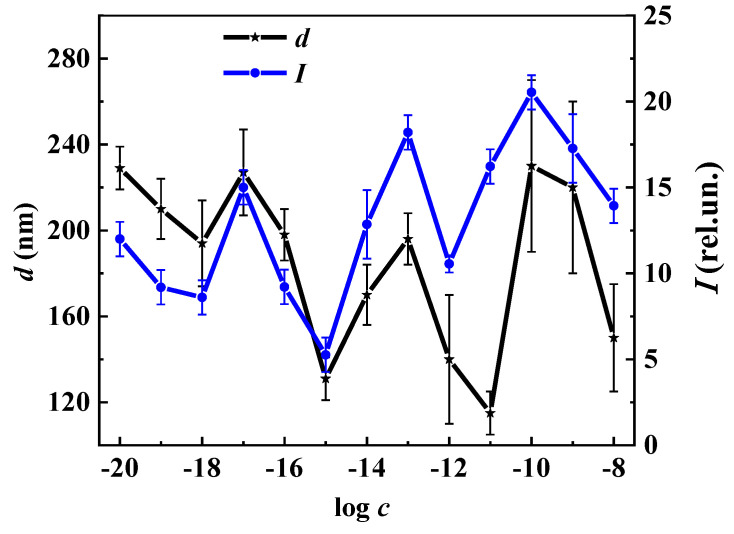
Dependence of the particles’ size (d), the fluorescence intensity (I) (λ_ex_ 225 nm, λ_em_ 340 nm) on the concentration (c, mol/L) of the *L*-Trp systems, 25 °C.

Figure 8 shows the non-monotonic concentration dependencies of the disperse phase size (d) and fluorescence intensity (I) (λ_ex_ 225 nm, λ_em_ 340 nm) of *L*-Trp systems, indicating a coherent change in the nanoassociate parameters (the relationship between *d* and ζ-potential is demonstrated in Figure 2) and their inherent ability to absorb and emit energy in the UV region in certain concentration intervals. Taking into account the found relationship between the parameters of nanoassociates, physicochemical properties, and fluorescence of the systems (Figure 2, Figure 3, Figure 4 and Figure 8, Supplementary Materials Figures S6 and S7), we can conclude that the previously established [29] pattern of the nanoassociate rearrangements’ determining role in the occurrence of coherent non-monotonic changes in the properties of disperse systems in the low-concentration range is also true for *L*-Trp systems.

Within the framework of the previously expressed hypothesis [29,36,37,38,39], it can be assumed that *L*-Trp systems are capable of affecting biological objects in a wide concentration range in which a dispersed phase is formed and non-monotonic changes in physicochemical properties and fluorescence intensity are observed. The degree and sign of the impact will depend on the properties of the dispersed systems at a particular dilution, as well as on the nature of the test objects. To check this assumption, we determined the effect of aqueous *L*-Trp systems on hydrobionts using the certified procedures for monitoring the toxicity of natural waters and wastewater [21].

**Figure 9:**
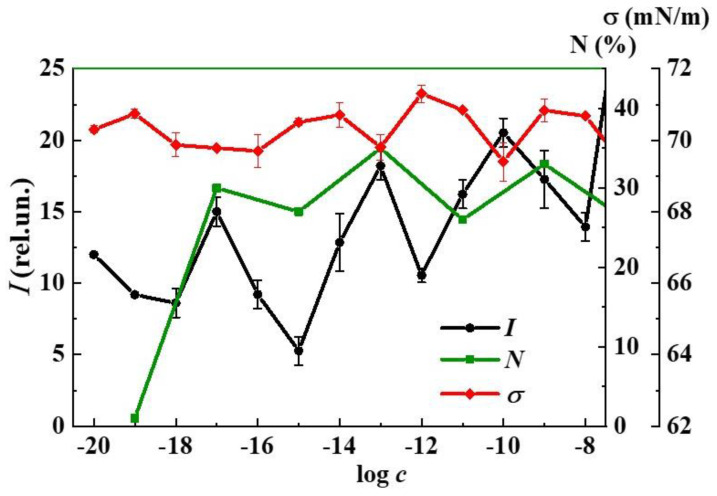
Dependence of the fluorescence intensity (I) (λ_ex_ 225 nm, λ_em_ 340 nm), surface tension (σ), and inhibition of the abundance of green algae *Chlorella vulgaris* (N) on the concentration (c, mol/L) of the *L*-Trp systems, 25 °C.

A study of the *L*-Trp systems effect on algae (*C. vulgaris*) showed that the presence of *L*-Trp in the test environment in the concentration range 1 × 10^−19^–1 × 10^−3^ mol/L has a harmful effect on green algae, consisting of non-monotonic inhibition of abundance (N) by 10–35% compared to control (Figure 9). The maximum reduction of algae abundance by 30%, 35%, and 33% occurs at 1 × 10^−17^, 1 × 10^−13^, 1 × 10^−9^, and 1 × 10^−7^ mol/L, respectively, which indicates the toxic effect on algae of the *L*-Trp systems at these concentrations [21].

The influence of *L*-Trp systems on infusoria (*P. caudatum*) in the concentration range 1 × 10^−19^–1 × 10^−3^ M is expressed to a lesser extent and consists in weak non-monotonic stimulation of their fertility by 10–20% compared to the control with maxima at 1 × 10^−17^, 1 × 10^−15^, 1 × 10^−13^, 1 × 10^−5^, and 1 × 10^−3^ mol/L, which is considered to be harmful [21].

Probably, similarly to some phytohormones, *L*-Trp in low concentrations can have an exogenous ecoregulatory effect on protozoa leading to changes in the numbers of different populations in their habitat [15,27]. Differences in the response of the test organisms used in the work to the presence of *L*-Trp in the aquatic environment are probably determined by their different levels of organization (algae and protozoa), and different types of cell structure, reproduction, and feeding methods.

When comparing the obtained data on the properties and bioeffects of *L*-Trp systems, conspicuous is the fact that toxic and harmful effects on hydrobionts occur in the vicinity of the abovementioned critical concentrations, where extreme changes in nanoassociate parameters and system properties are observed (Figure 2, Figure 3, Figure 4, Figure 8 and Figure 9). Studies [9,10,48] used organotypic cultivation of tissue fragments to establish that in the vicinity of some of the critical concentrations, i.e., at 1 × 10^−10^ mol/L and 1 × 10^−12^ mol/L, *L*-Trp systems affect cell proliferation of the cerebral cortex, subcortical structures, and rat liver, inhibiting or stimulating it by 35–31% compared to control.

The data shown in Figure 9 indicating the coherence of fluorescence and biological properties lend evidence to a previously stated suggestion that the observed fluorescence can be used to predict the bioeffects of highly diluted aqueous systems of BACs [36,37,38,39]. Considering such systems as simple models of natural water containing DOM, the obtained results can provide new information concerning fluorescence monitoring of DOM in surface water systems. In DOM fluorescence monitoring, the spectral band λ_em_ of 300–350 nm serves as a marker of the presence of tryptophan-like components or protein-like fractions in water [6,7,28]. The established correlation between fluorescence and nanoassociates’ parameters (Figure 2 and Figure 8) indicates that this band also reflects the presence of a dispersed phase in water, formed mainly by ordered structures such as EZ-water [35], and bioorganic substances of different structures in low concentrations, as a rule, below 1 × 10^−8^ mol/L. Consequently, this suggests that the natural water samples with a clearly expressed tryptophan-like region may contain not only tryptophan-like components, but also a dispersed phase of nanoassociate type. At the same time, the increase in fluorescence intensity (λ_ex_ 225 nm, λ_em_ 340 nm) in different samples will indicate an increasing probability of harmful effects of such water on hydrobionts by the mechanism of coordinated impact of the dispersed phase and changes in medium properties.

## 4. Conclusions

Thus, it is shown that aqueous solutions of the proteinogenic amino acid *L*-Trp in the calculated concentration range 1 × 10^−20^–1 × 10^−2^ mol/L are self-organized disperse systems with a disperse phase made of micelle-like particles and domains above the 1 × 10^−7^ mol/L threshold concentration, while below this concentration the disperse phase rearranges into nanoassociates of hundreds of nanometers in size, undergoing changes in size and negative ζ-potential as dilution proceeds. This is accompanied by a non-monotonic coherent change in the system’s physicochemical properties and fluorescence intensity.

We have established the interrelation between nanoassociates’ parameters (size, ζ-potential), physicochemical and fluorescent properties of the *L*-Trp system, as well as its influence on the growth and development of hydrobionts in the range of calculated concentrations 1 × 10^−19^–1 × 10^−3^ mol/L. The concentrations in the vicinity of c_th_, where the most coherent changes in nanoassociates parameters, specific conductivity, pH, surface tension, and fluorescence intensity of the system are observed, coincide with the concentrations at which the toxic effect on the cell abundance of *Chlorella vulgaris* green algae and harmful effect on the fertility of *Paramecium caudatum* infusoria are shown. Obtained results are consistent with the data from works [36,37,38,39,49], which establish a correlation between the fluorescence intensity of aqueous systems of some BACs at low concentrations and their effect on hydrobionts. The obtained data show that the observed fluorescence of such systems, which can be considered as a simple model of natural water, can be used in the monitoring of natural water samples to assess their harmful effects on hydrobionts.

## Data Availability

The original contributions presented in the study are included in the article/Supplementary Material, further inquiries can be directed to the corresponding author.

## Supplementary Materials

The following supporting information can be downloaded at https://www.mdpi.com/article/10.3390/nano12111792/s1. Figure S1: The correlation function (Coeff. corr.) and the volume particle size distribution (b) in *L*-Trp system at 1 × 10^−2^ mol/L; Figure S2: The correlation function (Coeff. corr.) and the volume particle size distribution (b) in *L*-Trp system at 1 × 10^−11^ mol/L; Figure S3: Dependence of the size of particles (d) and the specific conductivity (χ) on the concentration (c, mol/L) of the *L*-Trp, 25 °C; Figure S4: Dependence of the surface tension (σ) on the concentration (c, mol/L) of the *L*-Trp, 25 °C; Figure S5: Fluorescence spectra (λ_ex_ 225 nm, medium) of *L*-Trp systems, 25 °C; Figure S6: Dependence of the fluorescence intensity (I) (λ_ex_ 225 nm, λ_em_ 340 nm) and the pH on the concentration (c, mol/L) of the *L*-Trp, 25 °C; Figure S7: Dependence of the fluorescence intensity (λ_ex_ 225 nm, λ_em_ 340 nm) and the specific conductivity (χ) on the concentration (c, mol/L) of the *L*-Trp, 25 °C.
