# Supplementary material for: L-Tryptophan Aqueous Systems at Low Concentrations: Interconnection between Self-Organization, Fluorescent and Physicochemical Properties, and Action on Hydrobionts
Source: Nanomaterials (Basel). 2022 May 24;12(11):1792. doi: 10.3390/nano12111792 (PMC9182180; doi:10.3390/nano12111792)
Supplement: Supplementary file 1 [file nanomaterials-12-01792-s001.zip › nanomaterials-1644087-supplementary/nanomaterials-1644087-Supplementary Material.pdf]

Supplementary materials

L-Tryptophan Aqueous Systems at Low Concentrations: Interconnection between Self-Organization, Fluorescent and Physicochemical Properties, and Action on Hydrobionts

Irina S. Ryzhkina [1,*], Lyaisan I. Murtazina [1], Larisa A. Kostina [1], Diana A. Sharapova [1], Irina S. Dokuchaeva [1], Svetlana Yu. Sergeeva [1], Kristina A. Meleshenko [1] and Andrew M. Petrov [2]

[1] Arbuzov Institute of Organic and Physical Chemistry, FRC Kazan Scientific Center, Russian Academy of Sciences, 8 Arbuzov str., 420088 Kazan, Russia; limurt@yandex.ru (L.I.M.); lar.kostina2116@yandex.ru (L.A.K.); diana777_94@mail.ru (D.A.S.); 183561@mail.ru (I.S.D.); sergeevas@iopc.ru (S.Y.S.); kmeleshenko@inbox.ru (K.A.M.)

[2] Institute for Problems of Ecology and Mineral Wealth Use of Tatarstan Academy of Sciences, 28 Daurskaya Str., 420087 Kazan, Russia; zpam2@yandex.ru

* Correspondence: irina.s.ryzhkina@mail.ru or ryzhkina@iopc.ru

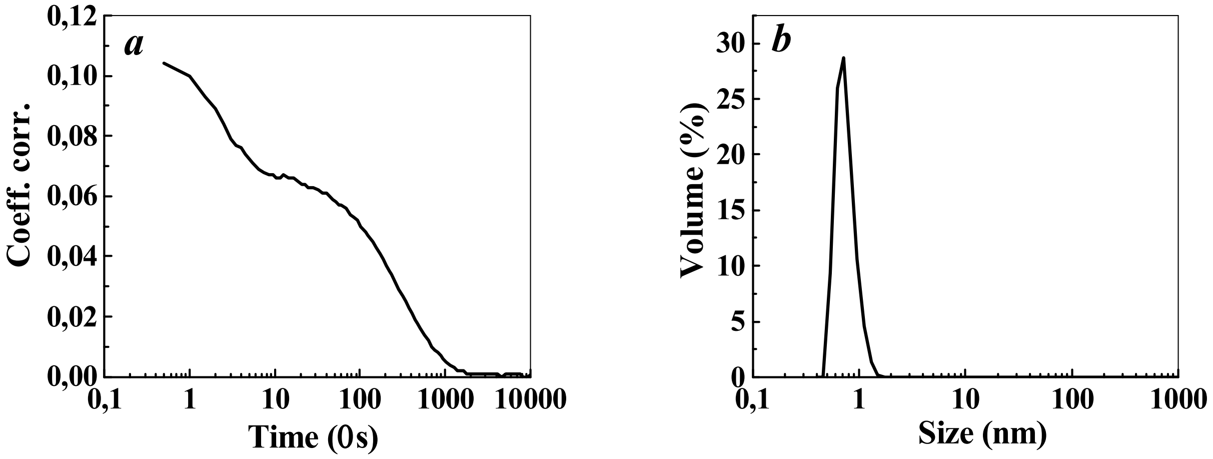

Figure S1. The correlation function (Coeff. corr.) and the volume particle size distribution (*b*) in *L*-Trp system at 1×10^{-2} mol/L.

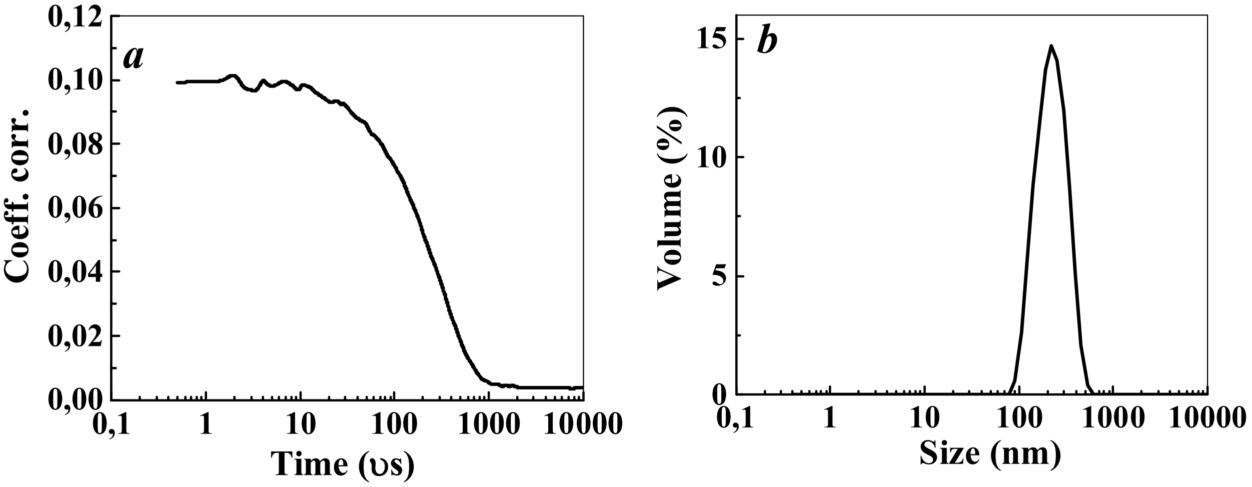

Figure S2. The correlation function (Coeff. corr.) and the volume particle size distribution (*b*) in *L*-Trp system at $1{\times}10^{-11}$ mol/L.

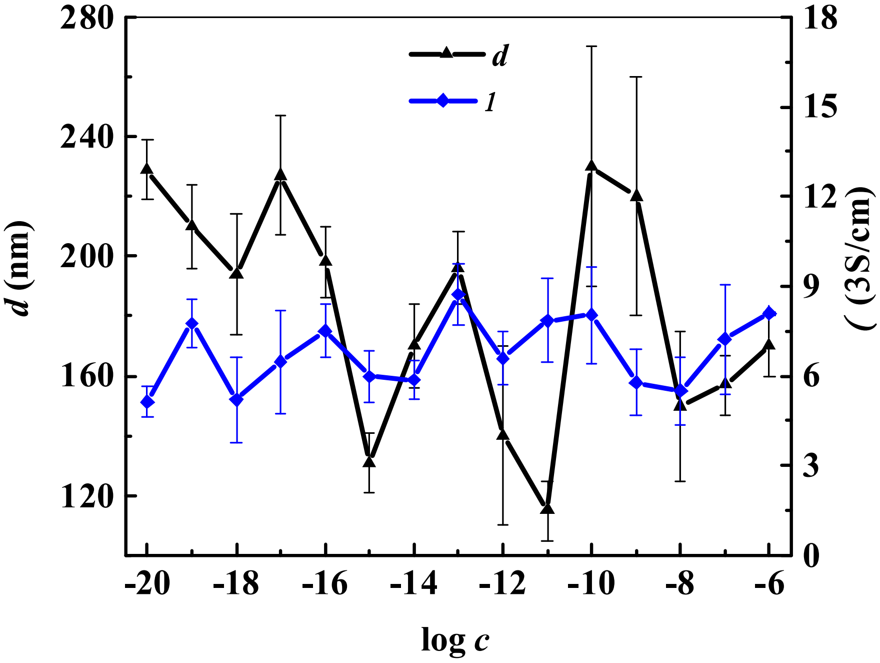

Figure S3. Dependence of the size of particles (*d*) and the specificconductivity (χ) on the concentration (c, mol/L) of the *L*-Trp, 25 °C.

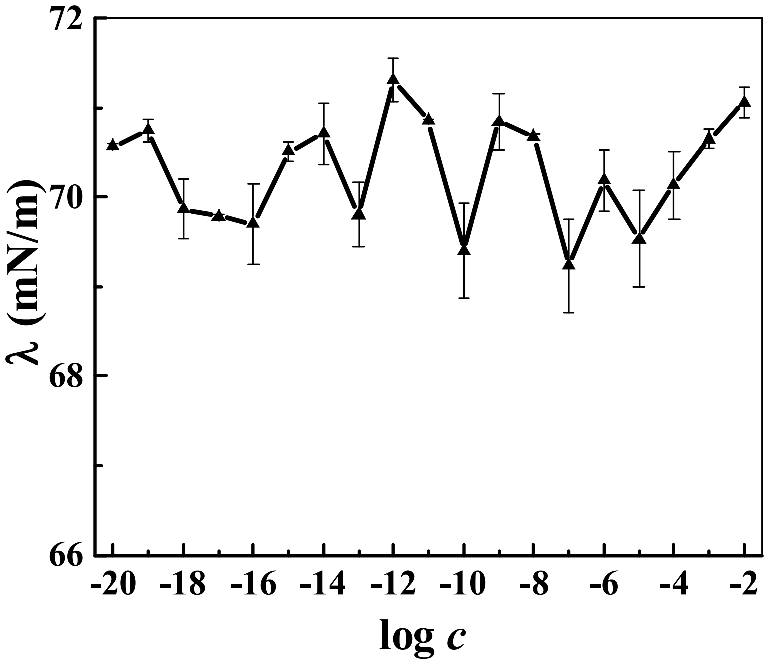

Figure S4. Dependence of the surface tension (σ) on the concentration (*c*, mol/L) of the *L*-Trp, 25 °C.

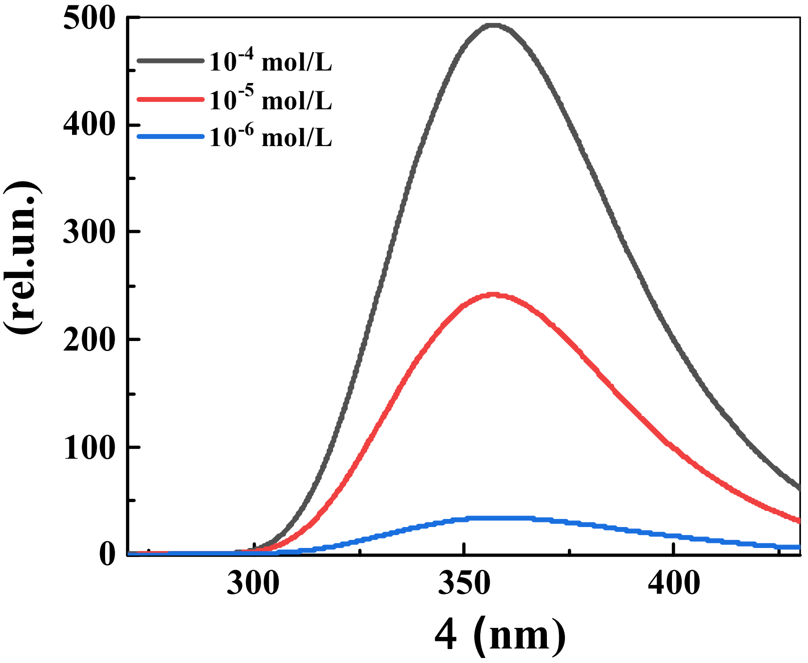

Figure S5. Fluorescence spectra (λ_{ex} 225 nm, medium) of *L*-Trp systems, 25 °C.

It is known [2,3] that in the range of high *L*-Trp concentrations (1 × 10^{-4} mol/L and higher), concentration quenching is observed. In the range of 1 × 10^{-5} – 1 × 10^{-6} mol/L the fluorescence intensity decreases linearly with decreasing *L*-Trp concentration. As shown in [36–39], at concentrations around 1 × 10^{-7} mol/L and below, i.e. below the threshold concentration (see the text of the paper), the fluorescence intensity changes non-monotonically with dilution, which is related to the formation and rearrangement of nanoassociates capable of emission in the high dilution range of aqueous systems.

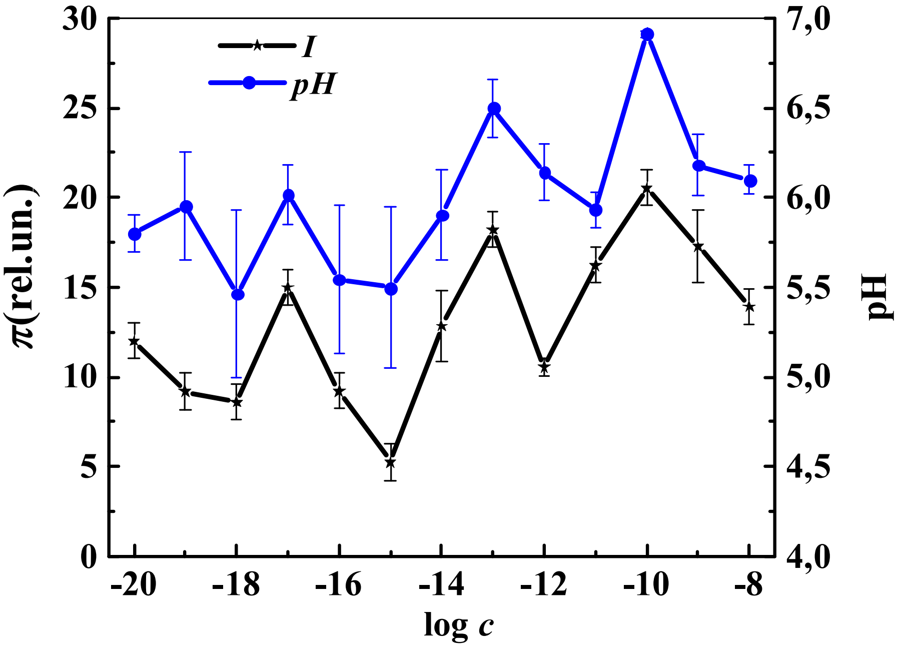

Figure S6. Dependence of the fluorescence intensity (*I*) (λ_{ex} 225 nm, λ_{em} 340 nm) and the pH on the concentration (*c*, mol/L) of the *L*-Trp, 25 °C.

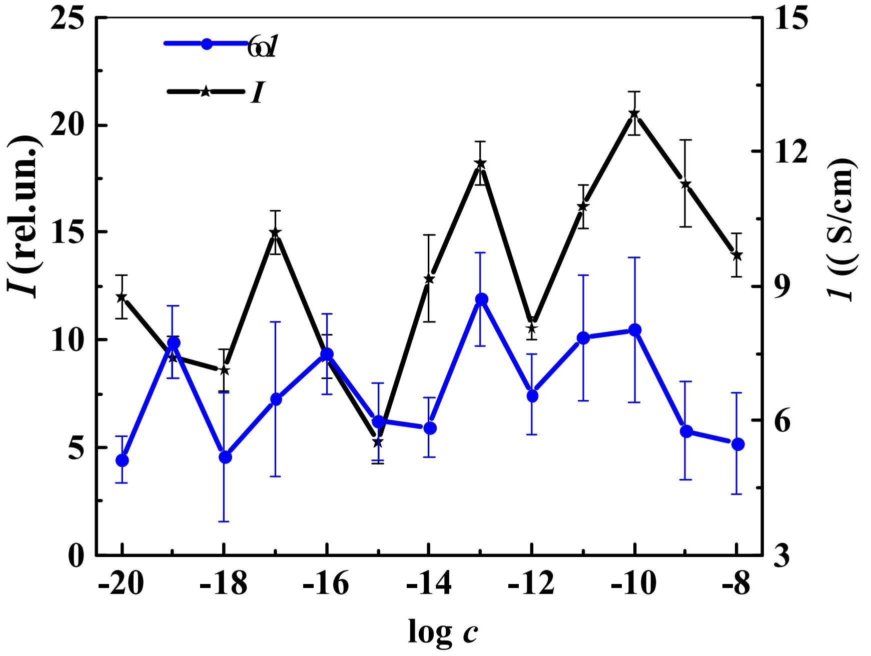

Figure S7. Dependence of the fluorescence intensity (λ_{ex} 225 nm, λ_{em} 340 nm) and the specific conductivity (χ) on the concentration (*c*, mol/L) of the *L*-Trp, 25 °C.
